# The Mindfulness App Trial for Weight, Weight-Related Behaviors, and Stress in University Students: Randomized Controlled Trial

**DOI:** 10.2196/12210

**Published:** 2019-04-10

**Authors:** Lynnette Nathalie Lyzwinski, Liam Caffery, Matthew Bambling, Sisira Edirippulige

**Affiliations:** 1 University of Queensland School of Medicine Centre for Online Health Woloongabba Australia

**Keywords:** mobile applications, mindfulness, body weight, feeding behavior, exercise, stress, psychological, students

## Abstract

**Background:**

University students are at risk of weight gain during their studies. Key factors related to weight gain in this population include unhealthy weight-related behaviors because of stress. Mindfulness holds promise for weight management. However, there has not been any previous trial that has explored the effectiveness of a student-tailored mindfulness app for stress, weight-related behaviors, and weight. There is limited evidence that current mindfulness apps use evidence-based mindfulness techniques. A novel app was developed that combined evidence-based, mindfulness-based stress reduction and mindful eating (ME) techniques that were tailored to university students, with student-relevant themes for targeting weight behaviors, weight, and stress.

**Objectives:**

The aim of this study was to test the effectiveness, acceptability, and feasibility of a student-tailored mindfulness app for weight, weight-related behaviors, and stress. Testing this app in a rigorous randomized controlled trial (RCT) for these outcomes is a novelty and contribution to this emerging field.

**Methods:**

A 2-arm RCT of an 11-week duration was undertaken at the University of Queensland. Students were either randomized to the mindfulness app (n=45) or to a behavioral self-monitoring electronic diary (e-diary; n=45) for diet and exercise. Analysis of covariance was used to compare differences in weight, stress, mindfulness, ME, physical activity, and eating behaviors between both groups.

**Results:**

Neither the mindfulness app group nor the e-diary group lost weight and there were no differences between the groups at follow-up. The mindfulness app group had significantly lower stress levels (*P*=.02) (adherers only), lower emotional eating (*P*=.02), and uncontrolled eating (*P*=.02) as well as higher mindfulness (*P*≤.001) and ME levels overall (*P*≤.001). The e-diary group had higher metabolic equivalents of moderate activity levels (*P*≤.01). However, the effect sizes were small. Regular adherence to mindfulness exercises in the app was low in the group. The majority of students (94%) liked the app and found it to be acceptable. Compared with other exercises, the most helpful reported meditation was the short breathing exercise observing the breath (39.4% [13/33] preferred it).

This was the first RCT that tested a mindfulness app for weight and weight-related behaviors in students. The modest level of user adherence likely contributes to the lack of effect on weight loss. However, there was a small, albeit promising, effect on weight-related eating behavior and stress.

**Conclusions:**

A mindfulness app demonstrated effectiveness for stress, eating behaviors, mindfulness, and ME, but the effect sizes were small. Future studies should be conducted over longer periods of time and with greater participant compliance.

**Trial Registration:**

Australian New Zealand Trial Registry ACTRN12616001349437; https://www.anzctr.org.au/Trial/Registration/TrialReview.aspx?id=371370 (Archived by WebCite at http://www.webcitation.org/761cc2K6ft)

## Introduction

### Background

Obesity and overweight are important international public health challenges that are critical to tackle across the globe given that they are leading risk factors for premature mortality and morbidity from a range of chronic diseases [1]. University students are a high-risk group for rapid and significant weight gain over a short period of time [2-5]. Recent reviews suggest that 60.9% of students gain weight in their freshman year [4]. The prevailing literature indicates that students who gain weight in their first year gain between 3.1 and 3.38 kg [3,4], and the reported rate of weight gain ranges from 110 to 156 grams per week [6,7]. This is significantly greater than the reported weight gain in young adults in the general population as the literature indicates that they gain anywhere between 605 grams and 1 kg per year [8-10]. Research also indicates that university students continue to gain weight during the remainder of their study years [11].

The leading causes of weight gain in this population have been reported to be an unhealthy diet, insufficient physical activity, and stress [3,5]. Stress has been reported to be highly prevalent in college students, with 80% experiencing some form of stress in their regular student lives [12,13]. The prevailing literature has linked stress with weight gain [14-17] and engagement in maladaptive weight-related behaviors in university students such as binge eating before exams, increased consumption of unhealthy food, or physical inactivity in students [18-30].

Emerging research suggests that mindfulness may hold potential for weight management and weight-related behaviors including reduced emotional and binge eating [31,32] as well as reduced stress [33]. The most recent systematic review and meta-analysis found that mindfulness assists with weight loss by 6.8 pounds, with moderate significant effect sizes (*g*=0.42; 95% CI excluding 0; *P*<.01), and that the effect size increased by 0.10 when both formal mindfulness practices were combined with informal practice [34]. They also found that the effect was greatest for weight-related eating behaviors than for weight (*g*=0.70; 95% CI excluding 0; *P*<.01) [34].

In addition, the most recent systematic review of mindful eating (ME) found that ME alone not only assists with weight management but also weight behaviors such as cravings and control of excess caloric intake [35]. Given that binge eating has been associated with weight and obesity [36,37], targeting these behaviors to promote healthy eating patterns and weight management is desired.

Mindfulness refers to a state of heightened awareness of oneself including one’s feelings, perceptions, and senses in a nonself-critical manner while blocking out any thought processes that act as hindrances to present moment mediation [38]. ME involves a heightened awareness of one’s bodily senses in response to the sight, taste, and sound of food as one eats by being attentive to one’s salivary, olfactory, and visual responses and ceasing to eat when one feels full [39]. Details of the types of mindfulness-based therapies are beyond the scope of this study, but in short, the main therapies include mindfulness-based stress reduction (MBSR), mindfulness-based cognitive therapy (MBCT), dialectical behavior therapy, and acceptance and commitment therapy (ACT) [40-42]. Traditional MBSR comprises 8-week therapy sessions that include Hatha Yoga, walking and sitting meditation, body scans, and assignments at home through self-reflections coupled with group sessions [40-42]. MBCT is similar to MBSR with the addition of cognitive therapy [40]. Note that this study will focus specifically on MBSR and ME as the intervention has been found in both.

Our recent systematic review (manuscript under peer-review journal) [43], which focused on university students [44-69], found that that there were positive associations between mindfulness and physical activity, which included self-efficacy, overall levels, and time spent engaging in physical activity, though there were few studies, and most were limited by being cross-sectional. The review also found some support for a positive association among mindfulness and healthy eating behavior and diet including reduced binge eating, emotional eating, and some support for improved dietary intake of fruit and vegetables and reduced fat [43]. However, the review [43] found that were few studies on dietary intake, most studies were limited by being cross-sectional, and more longitudinal studies are needed [44-69].

Our review additionally found that there were very few mindfulness-based randomized controlled trials (RCTs) for weight loss in university students and that more high-quality trials are needed in tandem with trials that adopt diverse mindfulness approaches such as MBSR [43]. Research also suggests that combining approaches rather than using 1 single approach such as MBSR and ME is more successful [31] along with the fact that both formal and informal practices result in the greatest weight loss as described earlier [34].

Given that potential mindfulness holds for assisting with weight management, adoption of healthy weight-related behaviors, and stress reduction, promoting mindfulness in college students is important. However, research indicates that accessibility and availability to weight management services are limited across campuses and students are placed on waitlists for various counseling services [70,71]. As accessibility to general counseling services and weight management services is limited [70,71], it may be deduced that accessibility to special therapies like mindfulness on campus would be equally difficult to gain access to as well, although studies are needed to specifically explore access to mindfulness training on campus. Nonetheless, students do have busy schedules; therefore, developing an intervention that may be easily accessible to them at any time and place irrespective of their student roster and social calendar would be very helpful.

One novel platform for delivering weight loss and weight-related behavior change interventions has been mobile health (mHealth), which includes the use of devices such as mobile phones personal digital assistants, tablets, and iPods [72]. mHealth holds numerous benefits, which include accessibility and portability to health care and promotion [73]. The author’s previous published meta-analysis of mobile devices for weight loss found that they are effective in assisting with weight loss as a moderate significant effect size is exhibited (Cohen *d*=0.43; *P*<.05) [74]. None of these interventions had a mindfulness focus [74].

To date, there has not been a mobile mindfulness-based intervention that assessed the effectiveness of a mindfulness-based app or mindfulness-based short message service (SMS) text messages for weight management and healthy weight-related behaviors as well as stress in university students. Our second recent systematic review [75] reviewed the types of electronic mindfulness-based interventions for weight, eating, and stress [76-96], finding that there has not been a mobile mindfulness-based RCT that implemented an app or SMS text messages for weight management or weight-related health behaviors (diet and exercise) in university students. We identified 1 web-based intuitive eating intervention [93], which did not lead to body mass index (BMI) changes, as well as a multipurpose mHealth app with ME as 1 component, not focusing on mindfulness as a central theme [88]. There has also not been a mindfulness-based app that is tailored to university students including those at risk of the Freshman 15, which integrates ME and MBSR techniques.

Since our review and trial protocol registration, there have been a few studies on mHealth and mindfulness including an ACT app for eating behaviors and another mindfulness app for improving weight-related eating behaviors and physical activity in teenagers [97,98]. One found that using food as a reward was reduced in the app group [97]. Although the study did not find that stress moderated this relationship, the techniques used were applied on the basis of ACT and not traditional MBSR, which would have been valuable for assessing if stress moderated the effects [97]. The other study was a mindfulness app pilot with videos, which included some components of formal mindfulness such as walking meditation and the body scan, along with ME, as well as physical activity and stretching techniques [98]. The study was tested in 15 teens, finding that it helped them with their reported ME, the duration of their engagement in mindfulness practice, awareness of eating behaviors, as well as physical activity [98]. There was also a recent mobile phone study that involved mindfulness phone coaching, which found that it assisted with weight loss and was particularly beneficial for those with maladaptive eating behavior such as emotional eating [99].

This is a very new field and this trial study will contribute to expanding the literature. The novelty of this study is the combination of MBSR and ME in a student-tailored app RCT for university students for not only weight-related behaviors and weight but also stress.

Given that both mindfulness and mHealth have been found to assist with weight [31,32,34,35,74] and that university students experience challenges with issues that these interventions address [3,4], combining the 2 in a mindfulness-based mHealth intervention for weight, weight-related behaviors, and stress in the university student population could hold great potential and is needed. By targeting stress, which is a determinant of maladaptive weight-related behaviors in college students [18-30], students may potentially benefit more than simply using an ME app that does not target the root cause. Similarly, targeting stress alone through MBSR without teaching ME may also be limiting as research suggests that combination approaches are most effective [31]. Thus, this app will integrate ME and MBSR techniques. Developing and testing a tailored mindfulness-based app that meets the unique needs of university students are novel, and the app may hold potential as a medium for health promotion in this population. Previous reviews have found that only 5% of mindfulness apps in iTunes are truly based on mindfulness, and our review of ME apps also found that they were weak [100,101]. Therefore, this app will integrate proven evidence-based MBSR and ME techniques.

### Aims and Objectives

The objective of this research was to develop and evaluate a mobile mindfulness-based app intervention that uses MBSR and ME techniques for weight loss, stress, and healthy weight-related behavior change in university students. The primary outcomes of interest are changes in weight and weight-related behaviors (eg, emotional eating and physical activity). Secondary outcomes of interest are changes in stress. Feasibility and acceptability will also be assessed.

Before the trial, the app was tested among the research group to ensure that its usability and functionality were intact. A subset of mindfulness-based messages had also been tested in a pilot exploratory qualitative pretrial study.

This study has the following main aims:

1. Determine if a novel mobile mindfulness-based app intervention is acceptable, feasible, and effective for weight loss and weight-related behavior change (eating behaviors and physical activity) when compared with a control receiving a self-monitoring electronic diary (e-diary) for diet and exercise with links to the World Health Organization (WHO) website
2. Determine if a novel mobile mindfulness-based app intervention reduces stress as a secondary outcome of interest when compared with the e-diary control

It is anticipated that the mindfulness app will be more effective for weight loss than the control e-diary as it targets stress-related weight behaviors in this specific population.

## Methods

### Participants and Setting

Students at the University of Queensland (UQ) at St Lucia and Herston campuses were recruited to participate in the Mindfulness App Weight Loss Trial of 11 weeks in duration. UQ is located in Brisbane, Australia.

### Recruitment

From July 10, 2017 to August 14, 2017, undergraduate students were recruited for the study. Recruitment strategies for the trial involved posters, flyers, social media, trial facts and UQ marketing and communications, and advertising via the website at UQ for the Centre for Online Health.

The recruitment material was categorized into general study information, which included posters and flyers on campus about the general study as well as detailed specific information that was in an information sheet. Trial facts advertised the information in a Web-based prescreener, and UQ marketing made a video about the trial and eligibility. The information sheet outlined the purpose of the study, the eligibility, the risks and benefits of participating, adverse event reporting, and rights to withdraw. Written informed consent was collected from participants before their enrollment in the study. This included consent to participate after participants read the information sheet with a clear description of the benefits, risks, including that the work will be published without releasing individual information. Participants were screened for eligibility in person and had signed the consent forms in person.

### Incentives

Participants received an Aus $20 Merlo Coffee voucher for their participation in the study. They were also given a number for an iPad mini draw at the end of the study.

### Eligibility

All healthy undergraduate students between 18 and 25 years of age who owned a mobile phone were eligible to participate if they wished to lose weight. Healthy was defined as anyone without a preexisting medical condition or history of serious psychiatric illness. Students <18 years or >25 years, pregnant women, and students with serious preexisting medical conditions were excluded. Students with a BMI<20 were also excluded. Students were asked if they had any serious medical conditions. Students with a preexisting medical condition were excluded.

### Randomization

A computer-generated random number sequence randomized eligible participants to an intervention comprising a mindfulness app or to a control group receiving a self-monitoring e-diary for diet and exercise. A parallel simple randomization 1:1 sequence was undertaken.

Allocation was concealed to students before their entry into the mindfulness-based weight loss study, and a central random number generation method was used to conceal allocation from the principal investigator LNL and the research team. An independent statistician not involved with the study from the Centre for Online Health conducted the randomization process. Participants were notified of the group to which they were assigned, with instructions on downloading the app if assigned to the intervention.

### Intervention Group: Mindfulness App

The intervention group received the mindfulness app (called My Student Mindfulness App), which contains ME and MBSR techniques. The app has been tailored to college students, with key themes on weight gain in college students (Freshman 15) and common college student stressors. Note that students tailored in this study are defined as being tailored specifically to the university student population, with student-relevant themes throughout rather than individual personal tailoring. The app seeks to educate, remind, prompt, and motivate students to practice ME [102] and stress reduction techniques [41]. MBSR techniques and meditation include body scan, diaphragmatic breathing, observing the breath, loving kindness meditation, concentration meditation, choiceless awareness mindfulness meditation, and Hatha yoga, which were adopted from key mindfulness books including elements from John Kabatt Zinn’s MBSR program [41]. Several books were consulted to teach ME including those which cover mindfulness [103-106] and also specialist books on ME [103-108]. In brief, the app has audios and videos in addition to standard written educational content. It also has tailored practical tips and advice for achieving a mindful lifestyle and advices on addressing barriers and promoting facilitators to meditation when in university. Another feature that was added to the app was mindful exercise that focused on encouraging physical activity and movement from a mindfulness perspective [105].

The app home screen shot is attached in Multimedia Appendix 1. The key features of the app and the specific MBSR and ME techniques used are summarized in Multimedia Appendices 2 and 3. The app had organized written lectures and audios in order of difficulty and similarity of topics. For example, students were first introduced to the simple breathing exercises in the audios and articles before progressing gradually toward the more difficult and lengthy meditation techniques. Although students were informed that they could gradually learn how to meditate at their own pace, they were sent a supplementary document as a suggestion about how to use the app with recommended activities that they could work their way up to. This is attached as a supplementary multimedia file in Multimedia Appendix 4. The app also has push notification enabled functions. The push notifications aim to educate, prompt, and remind students to practice mindfulness. The messages were sent during eating times to maximize ME opportunities.

### Control Group: E-Behavioral Self-Monitoring Diary

The control group received an electronic self-monitoring diary for its diet and exercise. The diary also had self-reflection space for students to think about key barriers they experience. They were also given links to WHO’s dietary and physical activity guideline information in the e-diary.

#### Assessments and Measures

Participant characteristics and demographic information were collected at baseline. Anthropometric measures were assessed at 2 time points at baseline and at 11-week follow-up objectively by the candidate and volunteers. Height was self-reported. Weight was measured using a digital scale. Standard procedures were used to assess weight, which required participants to remove their shoes before being weighed. BMI was calculated as kg/m^2^.

Physical activity was assessed using the International Physical Activity Questionnaire (IPAQ), comprising 27 questions on physical activity in the past week (IPAQ) [109]. A review of its validity and reliability across 12 countries found that it is both valid and reliable [109]. Previous college student weight gain intervention studies have used the IPAQ to assess physical activity [88]. The short version [110] was used to minimize the time burden on students given all the other questionnaires they had to complete. Eating behavior was assessed using the 3-Factor Eating Behavior Questionnaire as it assesses cognitive restraint, emotional eating, and uncontrolled eating, and it has been found to be valid and reliable for these measures in adults [111]. Stress was assessed using the Perceived Stress Scale, a 10-item self-reported survey of perceptions of stress [112,113]. It has been previously validated and found to be reliable for assessing stress in college students in a study across 3 colleges [114]. Mindfulness was assessed using the Cognitive and Affective Mindfulness Revised Scale, which has been previously validated [115]. ME was assessed using the Mindful Eating Questionnaire (MEQ), which comprises 28 items that assess eating behaviors (68). The MEQ has been validated previously in college students [116].

### Feasibility and Acceptability

Feasibility was assessed on the basis of participant retention and adherence. Participants were given a brief survey (Likert scale) upon completion of the study. It assessed adherence by asking participants how often they followed the app assignments through daily practice and whether they viewed all components of the app. The questionnaire asked whether they regularly used the app and practiced all the activities all the time, sometimes used it periodically, or did not use it at all.

The survey also assessed likability/acceptability of the intervention by assessing the degree of likability using a Likert scale. An extra questionnaire was added that asked about the most relevant meditation techniques used.

#### Ethics Approval

This study has been approved by the UQ Human Research Ethics Committee clearance number 2017000802).

#### Sample Size Calculation

As this is the first mobile mindfulness-based app intervention for weight and weight-related behaviors in university students, the anticipated effect size is not known. However, a sample size range may be estimated on the basis of certain assumptions. A previous mindfulness-based, Web-based stress reduction 2-arm RCT [89] estimated that a sample size of 50 in total would be needed for 80% power (alpha=.05) to detect a difference on the basis of a medium effect size in previous meta-analyses. The author’s previous mHealth weight loss meta-analysis also found a medium effect size [74]. Assuming a medium effect size using a 1-side hypothesis (one-tailed) at alpha=0.05 and 80% power to detect an effect if one exists, a sample size of 58 would be required when allowing for a 10% dropout (for a 20% dropout, the size would need to be N=66) according to a consultation with a statistician. A sample size twice this size (N=114) would be required if one assumes a medium effect size at a lower end of the medium effect size distribution. Hence, this study aimed to recruit no less than 58 participants, with the goal of at least doubling this number as the target (N=114). Note that 80% power to detect a difference if one exists refers to a difference between both the app and control group in the main outcome of interest, which is weight change. A 1-tailed hypothesis was used as the hypothesis was directional, whereby we hypothesized that the app would be superior to the control e-diary for weight change. The sample size estimates are based on previous mindfulness traditional interventions for weight in tandem with previous mHealth interventions for weight.

#### Enrollment

At the end of the recruitment, a total of 90 students who met eligibility were enrolled in the study.

#### Data Analysis

Participant baseline characteristics were summarized using descriptive statistics (means, percentages, and standard deviation). To ensure that randomization was conducted correctly and that there were no significant differences between groups on any baseline characteristics, differences between groups were tested using a general linear model and independent sample two-tailed *t* tests (where data were nonnormally distributed or did not meet assumptions and nonparametric tests were undertaken) [117-119]. Residuals of the covariates were plotted to ensure that the data were normally distributed in tandem with checking for high correlations among covariates and undertaking Levene test for equality of variances. When assumptions were violated, data were transformed [120,121]. Differences in weight between the intervention and control were assessed using analysis of covariance (ANCOVA) where baseline weight values will be covariates [122]. The rational for this use is that Vickers and Altman argue that it is the preferred method when comparing differences between 2 groups in trials from baseline to follow-up over analysis of variance as it controls for baseline differences in a variable among groups (imbalance), which may influence the outcome [122]. Post hoc tests were conducted if the *F* statistic in the ANCOVA was significant for between-group differences in weight [119]. Changes in the other outcomes of interest were also assessed using ANCOVA. Categorical data were analyzed using the Chi-square test. Data were analyzed using SPSS 20 [123,124].

#### Reporting

This study was reported using the consolidated standards of reporting trials (CONSORT) guidelines. This trial was prospectively registered with the Australia and New Zealand Clinical Trials Registry ACTRN12616001349437.

## Results

**Figure 1:**
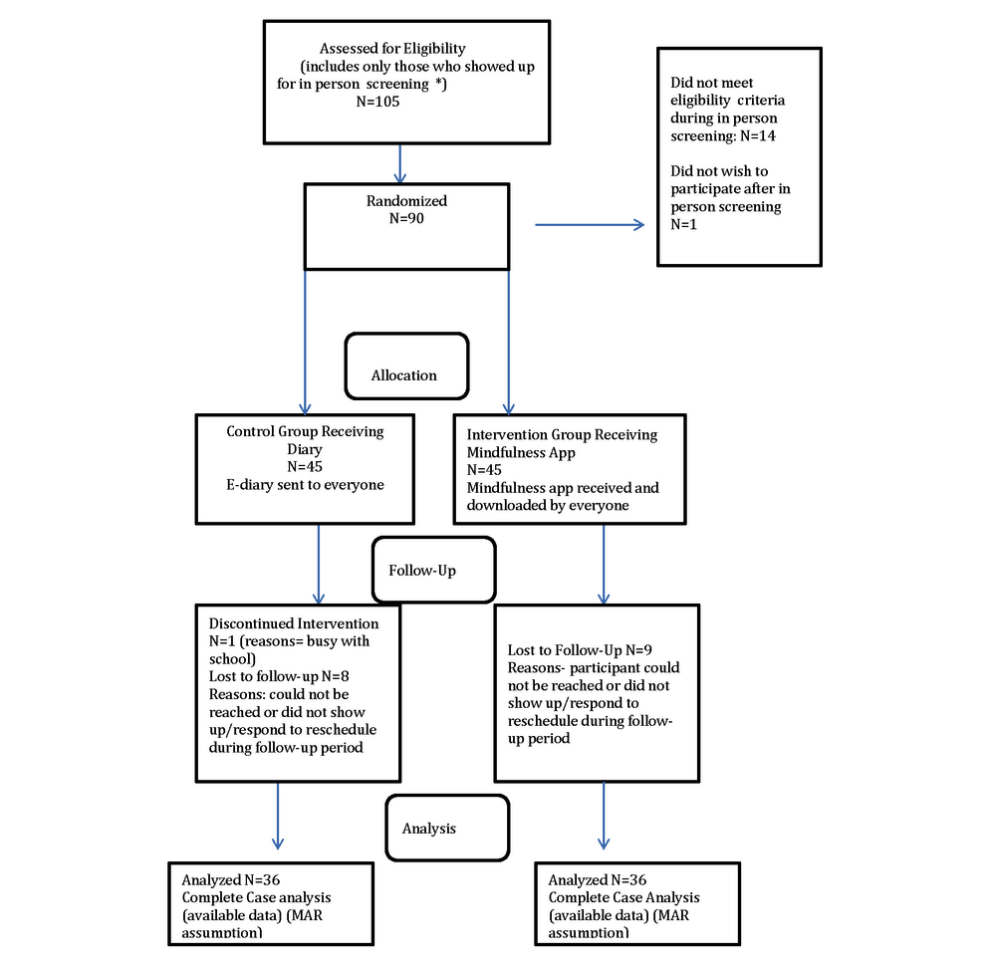
Consolidated standards of reporting trials flow diagram. MAR: Missing at Random.

The CONSORT diagram outlines the recruitment process and retention is illustrated in Figure 1. Participant characteristics including baseline demographic and descriptive information are summarized in Table 1 for the 90 participants enrolled (45 in each group). The mean weight of all study participants was 76.29 kg (range 53-126 kg). The mean weight in the intervention group was 76.7 kg and 75.5 in the control. The mean BMI in the intervention was 26.09 kg/m^2^ and 25.73 kg/m^3^ in the control. The groups were comparable on baseline characteristics, and there were no significant differences in weight between the groups or BMI. The intervention group had slightly higher stress levels at baseline. Changes in key outcome variables at 11-week follow-up are summarized in Table 2.

**Table 1:** Participant demographics and mean baseline anthropometrics.

Variable		Intervention app	Control e-diary	Total sample (N=90)
Age, mean; range		20.16	20.22	20.19; 18-24
Year		2.2	2.3	2.25=second year
Sex (female), %		74	61	67
Ethnicity (white), %		77	71	74
Weight, mean (SD)		76.4 (15.44)	76.18 (17.07)	76.29 (16.18)
Body mass index (kg/m^2^), mean (SD); range		26.09 (4.795)	25.73 (4.75)	25.91 (4.74); 21-43
Stress^a^, mean (SD)		18.18 (6.7)	18.82 (5.6)	18.5 (6.5)
Mindfulness^b^, mean (SD)		24.29 (4.326)	25.56 (4.031)	24.92 (4.326)
Mindful eating^c^, mean (SD)		2.588 (0.37)	2.639 (0.241)	2.61 (0.316)
**Weight-related behaviors^d^, mean (SD)**				
	Emotional Eating	7.78 (2.575)	6.64 (2.469)	7.21 (2.57)
	Uncontrolled Eating	22.02 (4.490)	21.58 (4.33)	21.80 (4.394)
	Cognitive Restraint	16.2 (3.9)	15.81 (3.8)	16 (3.87)
Physical activity^e^, total minutes per week		173 (105)	156 (105)	164.5 (105)

^a^Perceived Stress Scale.

^b^Cognitive and Affective Mindfulness Questionnaire.

^c^Mindful Eating Questionnaire.

^d^Three Factor Eating Behavior Questionnaire.

^e^International Physical Activity Questionnaire (short).

**Table 2:** Analysis of covariance for differences between groups in key outcome variables.

Variable	Measure	*F* test	*P* value	Mean difference (I-C)	95% CI	Partial eta squared	*R*^2^ (adjusted)
Weight	Digital scale	1.22 (df=1)	.27	0.76	−0.584 to 2.037	0.017	.971
		5.943 (df=1)^a,b^	.02^a,b^	−3.291^b^	−5.99 to −0.591^b^	0.090^b^	.326^b^
		2.775 (df=1)^c^	.10^c^	−2.151^c^	−4.727 to 0.425^c^	0.039^c^	.291^c^
Mindfulness	Cognitive and Affective Mindfulness Scale (revised)	14.580 (df=1)	<.001^a^	3.104^a^	1.482 to 4.726	0.174	.537
Mindful eating	Mindful Eating Questionnaire	21.035 (df=1)	<.0001^a^	0.295^a^	0.167 to 0.424	0.236	.402
Emotional eating	Three Factor Eating Behavior Questionnaire	5.893 (df=1)	.02^a^	−1.088	−1.98 to −0.194	0.079	.402
Uncontrolled eating	Three Factor Eating Behavior Questionnaire	5.974 (df=1)	.02^a^	−2.047	−3.717 to −0.376	0.080	.240
Cognitive restraint	Three Factor Eating Behavior Questionnaire	0.963 (df=1)	.330	−0.819	2.484 to 0.846	0.014	.290
MET^d^ moderate (min/week)	IPAQ^e^ (short)	7.885 (df=1)	.02^a^	−464^a^	−794 to −134	0.103	.113
Log transformed	IPAQ (short)	10.016 (df=1)	.01^a^	−4.07^a^	−0.671 to −0.143	0.278	.245
MET vigorous (min/week)	IPAQ (short)	0.737 (df=1)	.39	−306.933	−1020 to −406	0.394	.119
MET walk (min/week)	IPAQ (short)	0.095 (df=1)	.76	−76.150	−568 to 416	0.001	−.023
MET SUM	—	3.990 (df=1)	.05	−1006	−2011 to −1.296	0.055	.155
Log transformed	—	2.114 (df=1)	.15	0.127	−0.302 to −0.047	0.030	.103

^a^*P* value significant ≤.05.

^b^For adherers.

^c^Intention to treat.

^d^MET: metabolic equivalent.

^e^IPAQ: International Physical Activity Questionnaire.

### Weight Changes

There were no statistically significant differences in weight between both the mindfulness app intervention and control e-diary groups at follow-up using the ANCOVA (*P*=.27). Although neither group lost weight, analyses of between-group changes and paired sample tests (within-group changes) results indicate that neither the mindfulness app group nor the electronic behavioral self-monitoring diary control group gained any weight when comparing weight change from baseline to follow-up within and between groups (*P*>.05).

### Mindfulness

There were statistically significant differences in mindfulness between the mindfulness app intervention and control e-diary groups at 11 weeks using the ANCOVA (*F*=14.580(1); *P*<.01). After pairwise comparisons and adjustment for multiple comparisons using the Bonferroni correction, the intervention group had higher mindfulness levels by 3.104 on the mindfulness scale (*P*<.01; 95% CI 1.482-4.726; *R*^2^=.537; partial eta=0.174).

### Mindful Eating

There were statistically significant differences between both groups in ME levels at follow-up (*F*=21.035)(1); *P*<.01). The mindfulness app intervention group had higher ME levels by 0.295 points than the control e-diary group on the ME scale (*P*<.01; 95% CI 0.167-0.424; *R*^2^=.402; partial eta=0.236).

### Emotional Eating

It should be noted that at baseline, there were slightly higher levels of emotional eating in the mindfulness app intervention group compared with the control e-diary group (1.133; *P*<.04). However, ANCOVA controlled for these baseline differences, which may have influenced the outcome. The test on between-subjects effects using ANCOVA indicates that there is a significant difference between groups on emotional eating levels at follow-up (*F*=5.893 (1); *P*=.02). The pairwise comparison results indicate that the control group had significantly higher levels of emotional eating on the 3-Factor Eating Behavior Questionnaire by 1.088 points (*P*<.05; 95% CI −1.98 to −0.194), though the effect was small (adjusted *R*^2^=.402; partial eta squared=0.079).

### Uncontrolled Eating

The test on between-subjects effects using ANCOVA indicates that there is a significant difference between both groups on uncontrolled eating levels at follow-up (*F*=5.974(1); *P*=.02). The control e-diary group had higher uncontrolled eating levels than the intervention group by 2.047 points on the 3-Factor Eating Behavior scale (*P*<.05; 95% CI −3.717 to −0.376). Approximately 26% of the variability in uncontrolled eating levels is attributed to the intervention (*R*^2^ and partial eta=0.26).

### Cognitive Restraint

There were no statistically significant differences between both groups on cognitive restraint levels at follow-up (*P*=.33).

### Stress

The mindfulness app intervention group had lower stress levels than the control e-diary group, but this was not significant in the intention-to-treat (ITT) analysis. The per-protocol analysis (after removing those who did not use the app) and 1 outlier (the student was under severe mental distress because of personal family circumstances), there were significant differences in stress levels between the groups (*F*=5.943; *P*=.02). Pairwise comparisons indicate that the control had stress levels that were 3.291 points higher on the Perceived Stress Scale (PSS) than the intervention group (*P*<.05; 95% CI 0.591-5.992; *R*^2^=.326; partial eta squared=0.090).

### Physical Activity

#### Metabolic Equivalent Mod

There were significant differences between both groups in terms of metabolic equivalent (MET) moderate physical activity levels (*F*=7.885; *P*=.02). The control e-diary group had higher MET moderate activity levels than the control by 464.233 min/week after controlling for baseline levels (*P*<.05; *R*^2^=0.087; eta=0.103). However, Levene test for equality of variance did not meet assumptions, indicating that the results were less reliable. The residuals were also skewed. The data were log transformed and MET moderate vigorous physical activity levels remained significantly higher in the control e-diary group than in the app group (*F*=10.016; *P*=.01); *R*^2^=0.245; partial eta=.278) mean difference −0.407 (−0.671 to −0.143).

#### Metabolic Equivalent Vigorous

There were no statistically significant differences in mean MET vigorous activity levels between both groups at follow-up (*P*=.39).

#### Metabolic Equivalent Walk

There was no significant difference in MET walking levels between both groups (*P*=.76).

#### Metabolic Equivalent Total

There was a marginally significant difference between groups in the total MET level (*P*=.05). The control e-diary group had higher overall MET levels min/week by 1,007 min/week. However, the normality assumptions of the residual and Levene test for equality of variance were not met. The data were log transformed and there were no significant differences between groups (*F*=2.114; *P*=.15).

#### Differences in Physical Activity Level Categories

The Chi-square test was undertaken to test whether there were differences between the groups in the proportion of individuals in low, moderate, and vigorous physical activity categories. There were no significant differences between the mindfulness app intervention and control e-diary groups (*P*=.41).

#### Mindfulness Meditation Practice Exercises

Participants in the app group listed several mindfulness meditation exercises that were most useful (up to three). The most useful was the observation of the breath mediation exercise (39% [13/33] reported it being useful). The second most useful exercise was walking meditation (27 %, 9/33), which was found to be helpful. The third-most useful exercise was diaphragmatic breathing (21%, 7/33) and concentration meditation (21%, 7/33). A total of 12% (4/33) found the loving kindness meditation exercises to be useful. The least useful was the choiceless awareness mindfulness meditation exercise as only 3% (1/33) found it to be useful. A total of 9% (3/33) of students found all the exercises to be useful and 3% (1/33) did not find any of the exercises to be useful.

### Retention

A total of 72 out of 90 participants (80% retention) returned for the follow-up.

#### Acceptability/Likability

A total of 94% (32/34) of students reported that they liked the app overall and found it to be acceptable (either liked it, very much liked it, or somewhat), whereas 6% (2/34) did not like the app. Examples of mindfulness-based messages that were sent to students in the intervention are summarized in Textbox 1. The script is available in Multimedia Appendix 5.

#### Adherence

A total of 14% (5/34) of the students in the mindfulness app intervention group reported that they reviewed all the content and used it on a regular basis, whereas 61% (21/34) reported using it periodically. The remaining 23% (8/34) of students reported that they very seldom engaged with the app and did not review much of its content.

#### Feasibility

The app appears to be somewhat feasible as most students found it acceptable and the loss to follow-up was small. However, regular adherence was low.

**Sample mindfulness-based messages that were sent to the participants.**

Theme: stress

- What issues are on your mind at the moment? Identify any stressors and visually image that these stressors are like bubbles that you acknowledge by gently touching. Imagine these stressors dissipating like bubbles.

Theme: mindful awareness of body

- Try and take a mindful bite or spoonful for lunch/dinner today. What does it taste like, smell like, feel like, and sound like? Chew the food slowly. Mindfully stop when your body indicates you are full.
  Fun eating tip: Fill your bowl with fruit that includes all the colors of the rainbow. Really taste the differences.

Theme: mindful awareness of body

- A person takes 16 breaths per minute and 23,040 a day. Take a mindful breath, really focusing on the physical feeling of inhaling and exhaling and not thinking about anything else.

Theme: formal practice

- Long practice: Did you practice your body scan yet? Try practicing the body scan by tuning in with your body and how it is feeling from the head to the toe. Breathe in awareness to those areas.
  Short practice: Take a moment for a 3 min breathing space. Focus your attention on the breath, breathing as you naturally do in and out. Allow any thoughts, feelings, or sensations to enter your awareness. Gently hold them without judgment. Then gently return your attention to your breath.
  Walking meditation is a great way to connect with your body. Find some space and walk slowly, really feeling your movement.

Theme: informal practice or present moment awareness

- Having cues throughout the day can help you with being mindful. Students identified the lake as being a mindful moment spot. Go checkout the lake-the choice of the ideal mindfulness spot selected by students.

## Discussion

### Principal Findings

This study is important because it is the first RCT that has assessed the feasibility and effectiveness of a student-tailored mindfulness app for stress, weight management, and weight-related behaviors. As mentioned earlier, previous reviews critiqued commercial mindfulness apps for their lack of use of rigorous evidence-based, mindfulness-based techniques along with issues relating to quality of content and esthetics [100,101]. The app was novel as it combined MBSR evidence-based techniques with ME techniques that were tailored to students with special student-relevant themes throughout the app. The app was also novel because of the use of mindfulness-based messages for reinforcing weight loss, stress reduction, and healthy eating patterns as well as incorporation of mindfulness of exercise in the components.

In summary, the mindfulness app did not assist with weight loss over the semester and there were not any significant differences in weight between the mindfulness app intervention group and the behavioral self-monitoring e-diary group. The app assisted with some weight-related behaviors including emotional eating and binge eating as well as with increasing mindfulness, ME, and reducing stress in adherers. The e-diary group had higher moderate-to-vigorous physical activity levels.

Although the mindfulness app did not help with weight loss, students did not gain weight over the semester in both the app and the electronic behavioral self-monitoring diary groups. Behavioral self-monitoring is an already established method of weight management in the literature [125]. The fact that students who are a high-risk group for weight gain over the course of their semester did not gain weight suggests that both an e-diary for behavioral self-monitoring and a mindfulness app could potentially be used to prevent weight gain. Thus, the mindfulness app is comparable with an e-diary to prevent weight gain or ensure weight maintenance in students. More studies are needed to confirm this relationship. It would be of interest to study students entering their first semester of studies to see if these methods could be effective for preventing the Freshman 15 phenomenon or not. It is also important to study whether students should have the option of either type of intervention, a combined one, or a selection on the basis of their individual needs by exploring the stress versus motivation hypothesis for instance.

In addition to this, there were small albeit significant improvements in emotional eating and uncontrolled eating in the mindfulness app of the intervention group. This is in agreement with a recent mHealth ACT app trial, which found that there were increases in eating for physical rather than emotional reasons in study participants [97].

There was also a small effect of the app on stress in the per-protocol analysis, suggesting that the app has potential for assisting with stress and stress-induced eating behavior. The effect sizes were small, but it is likely that they relate to the low levels of regular adherence in the mindfulness app group. A previous study on mindfulness found that stress was reduced in those who adhered to the app for stress [81]. Addressing the problem of low regular adherence in this student population is needed. A further study is planned to better understand the barriers and facilitators of use to improve adherence to the app.

Since this study, there have been 2 studies in students using a mindfulness app for stress [126,127]. One 7-week intervention found that it significantly reduced stress [126], which is in agreement with this study, whereas the 4-week intervention found that it only improved emotional well-being [127]. It could be that the short length of the intervention, which is half the length of MBSR interventions [40], influenced the outcomes on stress, though the researchers mentioned that prespecified health issues may have influenced the result [127].

Early changes in eating behaviors and stress−related eating behaviors can take time before they have an effect on actual weight loss as maladaptive weight−related eating behaviors are indeed linked to weight gain and obesity [36,37]. Hence, it would be plausible that we may see weight loss in this population if studied over a longer period of time, especially if adherence will become more regular. It is also possible that it may take longer to observe weight change in mindfulness mHealth studies when compared with traditional behavior change mHealth studies as there have not been any previous comparisons in mHealth behavior change versus mHealth mindfulness apps for weight loss.

Mindful exercise was included as a module in the app with the aim of increasing physical activity. Mindfulness of exercise is an emerging field, which focuses on being present when one moves one’s body [105,128]. A previous systematic review found some evidence for higher physical activity levels in more mindful students than in their less mindful counterparts [43]. Overall physical activity increased in the mindfulness groups. However, the behavioral self−monitoring control e-diary group exhibited higher levels of MET moderate physical activity in min/week upon follow-up. This is consistent with previous findings as electronic self−monitoring has already been shown to increase physical activity levels in the literature [129,130]. It may be of research interest to develop a mindfulness app that combines elements of behavioral self-monitoring as our app only had a mindfulness practice self-monitoring diary rather than a diet and exercise self-monitoring diary.

In addition to this, we found that mindfulness levels and ME increased in the mindfulness app intervention group. These findings are in agreement with the findings of 2 mindfulness mHealth trials [98,99]. The recent pilot in teens, which used a mindfulness app, also found a high level of engagement in ME, but it was limited to being a pilot; therefore, there were no comparisons overtime and between groups [98]. The mindfulness phone coaching study also found that it helped with ME and mindfulness, though with only 1 mindfulness subset [99]. The study also did not find improvements in weight [99].

The most useful mindfulness meditation exercise was observing the breath, whereas the least useful was Choiceless Awareness Mindfulness Meditation. This makes sense as observing the breath is a very short 5-min exercise whereby one focuses on one’s breathing for up to 5 min in stillness [103,104]. It could be that simple, short meditation techniques are preferred in this population. Students also enjoyed walking meditation, which might seem to be practical on a green campus setting during breaks, as well as diaphragmatic breathing, which is longer than observing the breath and involves changing one’s breathing pace to a deeper one [104]. Other relevant exercises were concentration meditation and loving kindness, which are generally practiced for 15-30 min [104]. By contrast, the Choiceless Awareness Meditation is the most difficult to undertake according to Dr. Jon Kabat-Zinn as one must extend one’s awareness beyond the breath, to the body, and one’s thoughts without becoming attached to them [103,104]. This is practiced for up to 45 min [104].

In terms of feasibility, we found the mindfulness app to be somewhat feasible. The retention in the study was 80%, which is relatively high. Overall, students liked the app and found it acceptable. As mentioned earlier, more information is needed to find out ways to improve adherence by ensuring regular usage.

Another interesting part of this study involved the use of mindfulness-based SMS text messages, which was a novelty. There was 1 recent study that used these messages to encourage mindfulness practice in depressed patients, though the results were mixed as they did not work for all patients in terms of feasibility [131]. This study found that participants enjoyed the intervention, and more information on message perceptions will be published in an upcoming posttrial paper. More studies on mindfulness-based SMS text messages for weight are needed in the field.

### Strengths and Limitations

A strength is that this is the first RCT in the field to explore these relationships as our previous systematic review did not find such a study [75]. This study adds to the evidence base under the fields of mindfulness and mHealth (mobile mindfulness weight interventions). This is the first study that has also used mindfulness-based push notification messages. This study also measured mindfulness and ME, which is a strength over past studies [32] as it demonstrates that the app actually increased mindfulness.

A limitation is that this study was only for 11 weeks, and it may take longer to see the effects. However, there have been previous mHealth studies for weight loss that were of comparable duration [132,133] and still reported weight loss. Furthermore, traditional MBSR interventions are typically only 8 weeks in duration, indicating that this study’s duration was significantly longer [40,103]. Moreover, many mHealth MBSR interventions for stress were of similar short duration [75]. Two studies were only 1-month long and still found improvements in stress [96], with the latter finding improvement after the study [90]. The rationale for undertaking a study that was just under a semester in duration was to ensure that students would return for follow-up during the examination period as the holiday break to the new year could have potentially led to a significant loss to follow-up.

In addition, we did not have a standard no intervention control as originally planned as ethics had advised to offer 2 interventions for both groups. Furthermore, although we ensured allocation concealment, we did not ensure blinding. Ideally, study personnel as well as participants would be blinded to their intervention group [134]. It is possible that participants in both groups may have learned off each other. Another limitation involves the use of the *International Physical Activity Questionnaire*-Short Form, which has been found to be less reliable than the long form [135]. However, the short form was the most appropriate in our study given the sheer number of other measures.

Another minor limitation was receiving self-reported height rather than measuring height in person. However, students knew their height from their common IDs such as drivers’ licenses. Research also indicates that the BMI may not always be the best indicator of weight given that muscle increases BMI and that it does not assess fat [136]. Weight was objectively measured in person, which is the strength of this study.

In terms of randomization, it was established that simple parallel randomization would be appropriate for this study rather than stratifying for clinical risk factors, which may affect outcomes [137] in this healthy population. This method also did not require the statistician who undertook randomization to have access to participant study data characteristics, ensuring complete independence from the study.

We also did not use imputation methods such as Last Observation Carried Forward to estimate the analyses without any dropouts as part of the ITT; however, given the relatively high retention and the inherent limitations of these methods [138], we did not feel it was necessary. Moreover, according to the 4-point ITT strategy [139], complete case analysis that involves analyzing the available data in our type of study design and form of analysis is acceptable if we assume that the dropouts were missing at random [139].

### Future Directions

Future studies could further explore mHealth apps and mindfulness in relation to stress, weight, and weight-related behaviors over longer periods of time. In addition, we briefly explored push notification messages as well as mindfulness SMS text messages toward the end of the study that aimed to motivate students to use the app and practice their techniques. This has not been previously undertaken in any study. It would also be of interest to further conduct follow-up mindfulness-based SMS text message interventions for stress, weight, and weight-related behaviors such as simple observation of the breath audios sent as MPEG-1 Audio Layer 3 files with motivational mindfulness-based messages. It may also be of interest to combine traditional behavioral self-monitoring SMS text messages for weight and mindfulness-based messages.

### Conclusions

In summary, this is the first mHealth mindfulness app RCT that has assessed the effectiveness of a student-tailored mindfulness app for stress, weight-related behaviors, and weight when compared with traditional, electronic behavioral self-monitoring of diet and exercise. We did not find that the app was effective for weight loss. However, neither the mindfulness app intervention group nor the control e-diary group gained weight over the course of the semester. We also found that the mindfulness app significantly assisted with stress, emotional eating, and uncontrolled eating relative to the control, though the effect sizes were small. We also found that the app increased ME and overall levels of mindfulness. The e-diary control group had higher levels of moderate MET min/week activity levels than the app group. We conclude that the mindfulness app holds promise for weight-related lifestyle behaviors related to stress and stress eating, but more studies are needed to confirm these relationships. Low regular adherence was an issue in this study and more studies are needed that can explore whether these relationships will have greater effects when used on a regular basis. Focus group studies are needed to better understand key barriers and facilitators of usage. Longer studies are also needed to study whether stress-induced eating behavior changes may lead to weight loss over longer periods of time during a student’s studies. Furthermore, studies should examine whether a mindfulness app and electronic behavior self-monitoring via diary are effective measures for preventing weight gain, and possibly the “Freshman 15.”

## Appendix

Multimedia Appendix 1 App Home screenshot.

Multimedia Appendix 2 Mindfulness techniques in the app.

Multimedia Appendix 3 Mindfulness app media content.

Multimedia Appendix 4 App suggestions.

Multimedia Appendix 5 Mindfulness-based message options script.

Multimedia Appendix 6 CONSORT-EHEALTH checklist (V 1.6.1).

## Abbreviations

ACT: acceptance and commitment therapy
ANCOVA: analysis of covariance
BMI: body mass index
CONSORT: consolidated standards of reporting trials
e-diary: electronic diary
IPAQ: International Physical Activity Questionnaire
ITT: intention-to-treat
MBCT: mindfulness-based cognitive therapy
MBSR: mindfulness-based stress reduction
ME: mindful eating
MET: metabolic equivalent
MEQ: Mindful Eating Questionnaire
mHealth: mobile health
RCT: randomized controlled trial
SMS: short message service
UQ: University of Queensland
WHO: World Health Organization
