# Supplementary material for: The Mindfulness App Trial for Weight, Weight-Related Behaviors, and Stress in University Students: Randomized Controlled Trial
Source: JMIR Mhealth Uhealth. 2019 Apr 10;7(4):e12210. doi: 10.2196/12210 (PMC6479283; doi:10.2196/12210)
Supplement: Multimedia Appendix 1 [file mhealth_v7i4e12210_app1.pdf]

Table 1. Mindfulness techniques in the app.

Mindfulness practice and techniques	Type	Description	Media used to teach this	Duration
Observing the breath	Formal	Involves focusing on each inhalation and exhalation. Importance of not thinking about anything else.	Video demo, audios, and written lectures	A few min up to 5 min
Diaphragmatic breathing	Formal	Involves deeper and slower breathing. Full attention is paid to the breath	Brief video demo, audios, and written lectures	15 min
Sitting meditation	Formal	Includes concentration meditation exercises (several) and loving kindness meditation.	Audios and written lectures	20 min
		Concentration meditation focuses on a particular image that is empowering such as a waterfall for example and allows the participant to imagine being this image and overcoming anything that happens, resting still in awareness and breathing.		
		Loving kindness meditation involves beginning with love and good wishes at a singular level and extending it to friends, then even enemies, and the whole universe		
Walking meditation	Formal	Involves focusing on the breath and extending awareness to one’s movement including the movement of one’s feet, hips, and arms without deflecting one’s thoughts	Written lectures	20 minutes
Body scan	Formal	Involves focusing on one’s breath and extending awareness	Written lectures and audios	45 minutes

Author 2019-2-8 3:39 PM
Comment [1]: Please note that per journal style, a very long table must be uploaded as a Multimedia Appendix. Please delete the table from the manuscript and upload it as a Multimedia Appendix on the website with the caption. In addition, cite this table as a Multimedia Appendix in the body of text as applicable. The table citations must also be emended accordingly. If other Multimedia Appendices are cited in the manuscript, please ensure that all appendices are cited in a sequential order.

		to specific body parts beginning with one area such as a foot and working through the whole body. Any physical sensations are acknowledged.		
Choiceless awareness mindfulness meditation	Formal	Involves extending awareness beyond just the breath to other dimensions involving physical sensations, sounds in the environment, and any thoughts or feelings. While these feelings and thoughts are acknowledged, one does not engage with these thoughts and only notes their presence.	Written lectures and audios	45 minutes
Mindful eating	Informal	Involves eating slowly, responding to internal hunger signs (not external triggers), and using all five senses during the eating process (sight, sounds, smells, tastes, and physical sensations of the meal)	Article lectures, audios, and videos	Can involve one mindful bite for eating the entire meal and all meals mindfully
		It can be specially tailored for binge eating and craving control	Specially tailored audios in addition to general guided examples	
Mindful in general situations	Informal	Involves being present in everyday situations	Article lectures, but the focus was in the videos. The videos had a main focus on providing fun tips to be mindful in a student context on and off campus daily	Can be a few minutes of awareness to being aware in all situations throughout the day
Mindful exercise	Informal	Involves being present when exercising and being in tune with one's body	Video demos including swimming, tennis, and basketball	Can be a few minutes to a few hours depending on how long someone

				exercises or practices sport for

Adapted from Ref. (104)-(108).
