# Supplementary material for: The Mindfulness App Trial for Weight, Weight-Related Behaviors, and Stress in University Students: Randomized Controlled Trial
Source: JMIR Mhealth Uhealth. 2019 Apr 10;7(4):e12210. doi: 10.2196/12210 (PMC6479283; doi:10.2196/12210)
Supplement: Multimedia Appendix 2 [file mhealth_v7i4e12210_app2.pdf]

Table 2. Mindfulness app media content.

	Written lectures	Audios	Videos	Journal	Games
#1	Lecture 1. Introduction. University student stress and lifestyle challenges. Covers key challenges that are common for students	Audio1. Observing the breath	Video 1. Intro on stress, lifestyle, and weight in university students	Entry 1. Formal and informal practice	Game 1. User enters their stress and presses the bubble to watch it pop and disappear
					Game 2. Common issues students face with cloud solutions on how to mindfully address them
#2	Lecture 2. The relationship between stress, lifestyle, and weight in students. Discusses research	Audio 2. Diaphragmatic breathing	Video 2. What is mindfulness?	Daily formal and informal practice	Repeat of game options
#3	Lecture 3: Healthy lifestyle guidelines	Audio 3. Loving kindness meditation	Video 3. Mindfulness qualities	Daily formal and informal practice	Repeat of game options
#4	Lecture 4. Mindfulness intro: What is mindfulness?	Audio 4. Body scan	Video 4. Mindfulness of breath	Daily formal and informal practice	Repeat of game options
#5	Lecture 5. Relevance of mindfulness to students: In context	Audio 5. Mindful eating coconut meditation	Video 5. Common barriers to formal mindfulness	Daily formal and informal	Repeat of game options

Author 2019-2-9 10:54 PM
Comment [1]: Please note that per journal style, a very long table must be uploaded as a Multimedia Appendix. Please delete the table from the manuscript and upload it as a Multimedia Appendix on the website with the caption. In addition, cite this table as a Multimedia Appendix in the body of text as applicable. The table citations must also be emended accordingly. If other Multimedia Appendices are cited in the manuscript, please ensure that all appendices are cited in a sequential order.

			meditation practice	practice	
#6	Lecture 6. Qualities to avoid: Mindfulness barriers	Audio 6. Binger trigger meditation	Video 6. Mindful eating	Daily formal and informal practice	
#7	Lecture 7. Mindfulness qualities to foster	Audio 7. Hunger meditation	Video 7. Mindful fun eating	Daily formal and informal practice	
#8	Lecture 8. What do these qualities mean to me as a student?	Audio 8. Satiety meditation	Video 8. WHO dietary guidelines		
#9	Lecture 9. Case study of a mindful versus not mindful student	Audio 9. Sitting meditation (concentrative): Cave meditation	Video 9. WHO physical activity guidelines	Daily formal and informal practice	
#10	Lecture 10. Mindfulness formal technique modules	Audio 10. Sitting meditation (concentrative) lighthouse meditation	Video 10. Mindful exercise	Daily formal and informal practice	
#11	Lecture 11. Observing the breath : 10 breaths	Audio 11. Sitting Meditation (concentrative) sky meditation	Video 11. Mindful swimming	Daily formal and informal practice	
#12	Lecture 12. Diaphragmatic breathing: 15 minutes	Audio 12. Sitting meditation (concentration) Audio 12. Forest fire meditation	Video 12. Mindful tennis	Daily formal and informal practice	
#13	Lecture 13. Student centered	Audio 13. Choiceless	Video 13. Adopting a	Daily formal	

	tips/campus tips for mindful breathing	awareness mindfulness meditation	mindful lifestyle (barriers to a mindful lifestyle)	and informal practice	
#14	Lecture 14. Body scan: 45 minutes	Review Audios	Video 14. Tips for being mindful on and off campus	Daily formal and informal practice	
#15	Lecture 15. Sitting meditation: loving kindness		Video 15. Environmental cues	Daily formal and informal practice	
#16	Lecture 16. Concentrative meditation (sitting): Different exercises	Video 16. Loving kindness		Daily formal and informal practice	
#17	Lecture 17. Mindfulness awareness meditation: 5-45 minutes can increase by 5 min each week	Review audios	Review videos	Daily formal and informal practice	
#18	Lecture 18. Tips when practicing formal choiceless awareness mindfulness meditation			Daily formal and informal practice	
#19	Lecture 19. Walking meditation			Daily formal and informal practice	
#20	Lecture 20. Mindful eating what is it			Daily formal and informal	

				practice	
#21	Lecture 21. Mindful eating case study			Daily formal and informal practice	
#22	Lecture 22. Mindful eating tips			Daily formal and informal practice	
#23	Lecture 23. Making mindful eating fun/playing with one's senses mindfully			Daily formal and informal practice	
#24	Lecture 24. Mindful eating meditation			Daily formal and informal practice	
#25	Lecture 25. Binge trigger meditation			Daily formal and informal practice	
#26	Lecture 26. Hunger meditation			Daily formal and informal practice	
#27	Lecture 27. Satiety meditation			Daily formal and informal practice	
#28	Lecture 28. Mindful exercise			Daily formal	

				and informal practice	
#29	Lecture 29. Mindful exercise alone and in context			Daily formal and informal practice	
#30	Lecture 30. Mindful exercise in a group context			Daily formal and informal practice	
#31	Lecture 31. Mindful lifestyle barriers			Daily formal and informal practice	
#32	Lecture 32. Overcoming barriers to a mindful lifestyle: Case study of a mindful student			Daily formal and informal practice	
#33	Lecture 33. Being in the moment in the real world on and off campus			Daily formal and informal practice	
#34	Lecture 34. Student context: What students /peers recommend for being mindful on campus			Daily formal and informal practice	
#35	Lecture 35. Environmental mindfulness cues on and off campus			Daily formal and informal practice	

#36	Lecture 36. Formal mindfulness meditation barriers			Daily formal and informal practice	
#37	Lecture 37. Mindful yoga			Daily formal and informal practice	
#38	Lecture 38. Mindfulness informal and formal technique journal			Daily formal and informal practice	
#39	Poem 1. Renewal poem			Daily formal and informal practice	
#40	Poem 2. Mindfulness for students' poem			Daily formal and informal practice	
#41	Poem 3. Breathe poem			Daily formal and informal practice	
#42	Poem 4. Love and kindness poem			Daily formal and informal practice	
