# Supplementary material for: The Mindfulness App Trial for Weight, Weight-Related Behaviors, and Stress in University Students: Randomized Controlled Trial
Source: JMIR Mhealth Uhealth. 2019 Apr 10;7(4):e12210. doi: 10.2196/12210 (PMC6479283; doi:10.2196/12210)
Supplement: Multimedia Appendix 3 [file mhealth_v7i4e12210_app3.pdf]

Suggestions for Using My Student Mindfulness APP

My student mindfulness app is meant to teach you both formal meditation techniques and informal mindfulness practice where you are essentially present in your everyday lives on and off campus. It is meant to help you with managing your stress and teaching you how to adopt a healthy mindful lifestyle including mindful eating.

Questions and Answers

Q. How do I use the app?

A. The app is meant to be used by you at your own pace. We recommend using it on a daily basis. The articles, audios, and videos are arranged thematically with similar themes. Read an article and listen to the associated video and audio. The audios are very important for guiding you through your meditation practice as they teach traditional mindfulness techniques that are essential for your mindfulness practice. The videos are very brief and are meant to offer practical advice and tips around integrating mindfulness into your daily lives, with a greater focus on informal present moment awareness on and off campus. They are short and fun so be sure to check them out at your earliest convenience. The games are the least important and most of your time should be divided between the articles, audios, and videos (combination of all three, with the audios being given the priority).

Q How do I use the journal?

A: The Journal instructions are under the i Icon on the home screen. Tap on the help section. You are meant to log your formal and informal mindfulness practice in the journal. You can write about your challenges as well as your feelings. For instance if you have certain binge triggers or a feeling makes you want to eat, record this. Also record how often and how long you practice your mindfulness. You can also reflect on your informal present moment awareness by noting moments in the day when you were truly present and perhaps talk about photos you have taken of inspirational moments. I will not be collecting the journal so it is for your personal use entirely.

Q: What is Formal meditation?

A: Formal meditation techniques include the body scan, sitting meditation, walking meditation, and loving kindness meditation (Labee, E). Sitting meditation may be divided into any three exercises like practicing concentration meditation (imagining a lake, mountain, or other image that inspires you) , loving kindness (where you wish everyone happiness and wellbeing starting from yourself and extending this to your wider campus community and eventually the entire universe), and choiceless awareness mindfulness meditation where you allow 3 things to enter your awareness (physical feelings, sounds, and thoughts/emotions) by acknowledging them and not reacting to them (Labee, E). The third is considered the most challenging. You can begin formal meditation with very easy short breathing exercises like observing the breath where you focus on what it physically feels like

to breathe and try to not think about anything else. This can be done for even five minutes a day. Gradually increase you practice each week (Labee, E).

Q: How is Mindful Eating Different
A: Mindful eating is under informal practice, but it requires special attention to your sight, sense of taste, smells, and sounds when you eat your food slowly (Labee, E). Try and eat each meal with a mindful bite on and off campus, and gradually the aim of the app is to help you with becoming motivated enough for you to eat all of your meals mindfully (see modules for references and resources).

Q: What are some common tech issues?
A: Make sure your ringer is on (iPhone) otherwise you won't hear any sounds even if your volume is on max and you can listen to your music. If at anytime things freeze, restart your phone. The home screen (under i) has a help section so please go there if you have any questions and contact me if you experience any tech issues.

My Mindfulness Suggested Training Schedule: Techniques to be implemented daily during different times

Note: This is only a suggested schedule to gradually integrate the techniques and some of you may find that you wish to integrate these techniques by working through the modules sooner or reach the suggested time period of meditation sooner. The goal is for you to be well versed with mindful eating and have gained mindfulness-based stress reduction meditation skills and techniques. The goal also is for you to also find what formal techniques work best for you by trying a variety out. You can steadily increase your practice each week and become introduced to more advanced techniques at your own pace.

Week 1:
Intro to Mindfulness Module 1. General Overview of what mindfulness is, the benefits, and key qualities a mindful student has.
Observing the Breath Module: Practice doing 10 mindful breaths.
Slowly work your way up to 5 minutes a day over the first week. Listen to the observing the breath audio and read the observing the breath article.

Over the next two weeks increase your breathing exercise by 5 minutes extra each week until you have reached 15 minutes/day

Diaphragmatic Breathing Module
Begin with 5 minutes per day over week 1.
Increase each week by 5 minutes until you reach 15 minutes/day. Listen to the observing the diaphragmatic breathing audio. Read the diaphragmatic breathing audio. Watch the mindfulness of breath video.

Tasks for week 1 completed? Well done ☺
If not no worries, take a moment to commit to them now

Mindful Eating Modules: Begin week 1 by taking a mindful bite with a meal at least once a day. Work up to eating a full meal mindfully over the next week. Listen to the mindful eating video , mindful fun eating video, and mindful eating audio.

Informal Practice Modules: Be mindful throughout your daily encounters on and off campus by being fine-tuned to all your senses (sight, taste, smell, sound, and touch) whenever you experience anything. Be mindful in class, mindful of your campus environment, etc.

Week 2:
Mindful Eating Meditation: Practice the mindful eating meditation. Mindfully select your food items, mindfully prepare your meals, and eat mindfully. Try working your way to eating all of your meals mindfully.

Body Scan Module: Practice the body scan in week 2. Work your way up to 45 minutes per day –you can begin with however long you can and increase it each week by 5-10 min till you reach 45/minutes/day for 6 days over a period of at least 2 weeks. Listen to the body scan audio.

Informal Practice: Continue your daily informal mindfulness practice
Journal: Journal your mindful practice both formal and informal in the e-journal
Tasks for week 2 completed? Well done ☺
If not no worries, take a moment to commit to them now

Week 3: Listen to the binge trigger meditation audio in addition to your other practice
Continue with journaling your entries in the e-journal.
Tasks for week 3 completed? Well done ☺
If not no worries, take a moment to commit to them now

Week 4: Hunger meditation Module: Listen to the hunger meditation audio in addition to your other practice.

Week 5:
Satiety Meditation Module: Listen to the satiety meditation audio along with your other practice.
Continue your mindful eating and informal present moment awareness on and off campus.

Week 6:
Sitting Meditation Modules: Practice sitting meditation. Work your way through loving kindness, choiceless awareness, or concentrative meditation. You can alternate them , with the goal of reaching 45 minutes a day of sitting meditation. Remember you can begin sitting meditation with observing the breath or diaphragmatic breathing in a sitting

position for 5 minutes and gradually increase each week by 5 min up to 25 minutes per day.
Listen to the audios and focus your concentrating on a particular object representing strength and calmness such as the cave, mountain, lake, forest, or ocean meditation.

If you wish you can add this to your practice along with continuing with the formal practices described above: body scan, observing the breath, diaphragmatic breathing. Continue eating mindfully each day . Continue with journaling your entries in the e-journal
Tasks for week 6 completed? Well done ☺
If not no worries, take a moment to commit to them now

Week 7:
Walking meditation Module: Practice walking meditation daily for up to 15-25 minutes**.** Continue with your mindful eating and informal techniques.
Continue with journaling your entries in the e-journal.

Review any videos you have not watched as they are short and offer practical tips and advice for mindfulness barriers, mindfulness cues, making mindful eating fun, and adopting a mindful lifestyle on and off campus.

Repeat Core Exercises Weeks 8-12

- Emphasis on Mindful Eating Meditation with all meals and mindful selection of foods
- Depending on your personal needs, you can continue with the hunger/binge/satiety meditations
- Observing the Breath
- Diaphragmatic Breathing
- Body Scan
- Sitting meditation:(can be your choice of image concentration meditation such as the cave, lake, mountain , forest, or other inspirational image or an alternating combination with mindfulness awareness (attuned to your breathing, sounds, and thoughts that may enter your awareness field) with loving kindness-meditation
- Pick your favourite meditation techniques based on what worked for you thus far : if you prefer a type of sitting meditation technique like concentration meditation, then stick with what you like.
- Apply the 3 minute breathing space during the day as you see fit
- Continue applying mindfulness throughout the day informally through attention to all of your senses and what you are experiencing
- Continue journaling your entries
- Review any videos, audios, or article that need reviewing and make sure you have thoroughly viewed all of the app content

Reference
Labee, Elyse (2011). Psychology Moment by moment. A guide to enhancing your clinical practice with mindfulness and meditation. New Harbinger Publications.
