# Supplementary material for: The Mindfulness App Trial for Weight, Weight-Related Behaviors, and Stress in University Students: Randomized Controlled Trial
Source: JMIR Mhealth Uhealth. 2019 Apr 10;7(4):e12210. doi: 10.2196/12210 (PMC6479283; doi:10.2196/12210)
Supplement: Multimedia Appendix 5 [file mhealth_v7i4e12210_app5.pdf]

Push Notification Script of Message Options:
Theme: Stress: What issues are on your mind at the moment? Identify any stressors and visually image that these stressors are like bubbles that you acknowledge by gently touching. Imagine these stressors dissipating like bubbles.

Theme stress: Prioritizing your tasks and mindfully tackling them moment by moment can help with preventing stress.

Theme: Stress: Any time you feel stressed, practice your diaphragmatic breathing.

Theme: Mindful Awareness of Body: You have 206 bones in your body. Your foot has 26 bones and more than 100 muscles. Bring awareness to the feeling of your foot on the ground. What does it feel like? Breathe into the 26 bones in your right foot, and breathe out.

Theme: Mindful Awareness of Body: Sticks and stones may break your bones . Fun fact!? Did you know that the hand you use to write notes and type in class has 26, 34 muscles in your fingers , and 17 muscles in your palm? Being aware of your body including what it feels like can help with bringing mindfulness awareness to it. Now can you feel it in your bones literally?
Theme: Mindful Awareness of Body: How many muscles do you think you can flex in this very moment?

Theme: Mindful Awareness of Body: A person takes 16 breaths per minute and 23,040 a day. Take a mindful breath, really focusing on the physical feeling of inhaling and exhaling and not thinking about anything else.

Theme: Formal Practice: Have you been practicing your mindfulness techniques? Reflect on these experiences including sensations in your internal and external environment. Record any challenges in your journal.

Theme: Technique Fact: Subtheme Stress: Slowing down your breath with diaphragmatic breathing activates the parasympathetic nervous system, which in turn has a calming effect. it will slow your heart rate, lower your blood pressure, and promote relaxation by suppressing the flight or fight reaction to stressors both physical and psychological (Labe etc).

Theme: Formal Practice Education: Formal practice research suggests that slowing the breath stimulates the parasympathetic nervous system.

Theme: Formal Observing the Breath Practice: Mindfulness can be easily accessible to you at any time and place. All you have to do is observe your breath for a few minutes at your natural pace.

Have you been practicing your mindful breathing exercises? Gradually increase by 5 minutes each week.

Technique Fact: Ever experience your mind wandering when you practice formal mindfulness meditation? This is normal, gently bring your attention back to the breath each time.
Theme: Mindfulness Journal self-monitoring: Self-monitoring can help you keep track of practice and identify barriers or challenges. Reflect on them and record them in your e-journal.

Theme: Mindfulness Self-Monitoring and My Mindful Photo Moment: Try taking a photo of moments that inspires mindfulness in you both on and off campus or acts as a cue to be mindful. Reflect on them in your journal.

Theme: Formal Techniques Education : Mindfulness Choiceless Awareness Meditation Technique: Sitting in awareness by allowing thoughts to enter without reacting to them can be challenging. Any time you react to them, gently bring your awareness back to your breath.

Theme: Formal mindfulness Technique : Subtheme Body scan reminder: Did you practice the body scan yet? Try practicing the body scan by turning in with your body and how it is feeling from the head to the toe. Breathe in awareness to these areas.

Theme: Formal mindfulness technique :Subtheme Walking Meditation Knowledge: Walking meditation is a great way to connect with your body. Find some space and walk slowly, really feeling your movement.

Theme: Formal Mindfulness Technique: Loving Kindness Meditation: practice loving kindness meditation by sending good wishes to yourself, your loved ones, classmates, and the campus. Imagine this love growing and expanding.

Loving Kindness: Take a moment to send positive thoughts of loving kindness to yourself, your loved ones, your campus buddies, and the greater communities and globe.

Loving Kindness: Pay kindness forward. Smile to a new person on campus.

Theme Mindfulness Qualities: Reflect on the mindfulness qualities of acceptance, trust, non-judgmental attitude, and beginner's mind. Integrate these qualities in your daily interactions on and off campus.

Theme Beginner's mind: Try starting your day by seeing things like for the first time, with the beginner's mind of a child. You will be amazed at what you see. Observe the intricate beauty on campus.

Informal Practice Theme: Take a moment for a 3 minute breathing space. Focus your attention on the breath, breathing in as you naturally do and out. Allow any thoughts, feelings, or sensations to enter your awareness. Gently hold them without judgment. Then gently return your attention to your breath.

Informal Practice Theme: Morning wake up message: Morning lovely/beautiful. Take in a deep mindful breath of air. Breathe in the radiant warm energy of the sun. Breathe in the sun, breath out calm and love ☺

Informal Practice Theme: Morning wake up message: Start your day mindfully by really being present when you do usual things like getting ready for class, brushing your hair, teeth, washing your face, dressing up, and packing your backpack.

Informal Mindfulness of Sounds: Try listening to music which inspires mindfulness in you. It can be contemporary, classic, or nature.

Informal Mindfulness of Sounds: Listen to the sounds of water either through a CD, online, or going out in nature on campus.

Informal Mindfulness of Sounds: Students find that some songs can inspire mindfulness in them like Free Falling by John Mayer or Watch over me by Jason Fanning. Reflect on what songs inspire mindfulness in you and listen.

Theme: Informal Mindfulness of Smells: Buy flowers for your dorm. Really tune into your sense of smell and your sight.

Theme: Mindful Eating: Theme mindful drinking: Take a mindful coffee or tea break. Really smell the aromas and taste the flavours. Drink slowly.

Theme: Present Moment Awareness. Remember you only really have this moment and it is this moment that counts.

Theme: Present Moment Awareness: Remember being present in this moment of your student life shapes your next future moment.

Theme: Present Moment Awareness: When going out to school this morning, practice your mindfulness by really seeing, smelling, and feeling the environment around you including the natural landscape of your campus

Theme: Informal Mindfulness: Take a breath and vow to be mindful throughout your day from brushing your teeth to focusing on your lecture in the classroom setting to your meal setting.

Theme: Present Moment Awareness: Allow this message to be a cue for you to be in the moment. Now is your moment, embrace the present.

Theme: Present Moment Awareness: Subtheme: How to use senses mindfully on campus: A mindful student on campus tunes into their hearing by listening to sounds on campus and listen to their classmates and professor.

Theme: Present Moment Awareness: Subtheme: How to use senses mindfully on campus: A mindful student tunes into their sight on campus by really seeing the campus like the first time.

Theme: Present Moment Awareness: Subtheme: How to use senses mindfully on campus: A mindful student touches mindfully by doing simple things like feeling their pen and hand as they type in class.

Theme: Present Moment Awareness: Subtheme: How to use senses mindfully on campus: A mindful student uses their sense of smell on campus by smelling the natural aromas like flowers on campus, aromas in campus coffee shops, and perfume.

Theme: Present Moment Awareness: Did you know that mindful students really see, hear, feel, and smell the environment around them including the natural campus landscape? Being mindful isn't as hard and as dull, just simply feel the environment around you: look around, what does the natural landscape look like? Take a deep breath, can you smell what's in the air? Stop for a while and enjoy the harmony of the nature

Theme: Present Moment Awareness. The next time you are truly present, take a photo of something inspirational. It could be someone's smile or a photo of nature

Theme: Present Moment Awareness: Subtheme: Student context what students recommend: Having cues throughout the day can help you with being mindful. Students identified the UQ lake as being a mindful moment spot. Go checkout the lake –the choice of the ideal mindfulness spot selected by students!
Theme: Present Moment Awareness: Subtheme: Student context what students recommend: Consider taking a pause in your day by going to the UQ Lake. How many animals and plants do you see and which is your favourite?

Theme: Present moment awareness : Subtheme: Studies: Be mindful during your studies by avoiding multi-tasking and listening what your professor is saying, studying mindfully, and writing papers mindfully. Be mindful of areas you need to work on.

Theme: Present Moment Awareness: If you have a pet you can practice being mindful informally by mindfully petting your cat, dog, or caressing your bird.

Theme: Mindful eating Practice: Try taking a mindful bite or spoonful with each meal during the day. What does your food smell like, taste like, feel like, and sound like? Record your experience in your journal.

Theme: Mindful eating: Try changing the healthy foods you eat to avoid habitual and autopilot behaviours

Theme: Mindful Eating Treat: Treat yourself to a mindful tea break. Take several deep breaths, inhaling the aroma and tasting the tea. Know that it originated from a leaf from a land far away. Savour every bit with gratitude.

Theme: Mindful Eating: Have a mindful fun eating cooking date with friends by sampling different food items and really connecting with all five of your senses.

Theme: Mindful Fun Eating Tip: Fill your bowl with fruit that includes all the colours of the rainbow. Really taste the differences.

Theme: Fun Min Fun tip: Dr Pavel Somov recommends eating with a beginner's mind by having a friend offer food items while you are blind folded. This can be a fun way to get a heightened present moment awareness and to connect with one's taste buds.

Theme Mindful eating Binge/Craving: Anytime you experience cravings, remember to stop and assess if they are related to external stressors. Breathe in calm, and breathe out the craving. Imagine the craving is a cloud in the sky and you are a great vast sky, observing thoughts without reacting to them. The craving will pass like the clouds.

Theme Mindful eating: late night eating/binge: Anytime you have thoughts of binging at night or night/eating, breath in the craving, breath out calm.

Theme: Yoga: Take a moment this evening to practice some basic yoga movements, being mindful of your body's movement. Try child pose and cobra.

Theme: Mindful eating: A mindful eater is aware of their nutritional needs by combining their inner body's cues with their outer knowledge of nutrition. Have you been eating a balanced diet and a varied diet?

Theme: Mindful eating: Mindful eating involves tuning in with your senses by focusing on what the food tastes like, feels like, smells like, looks like, and even sounds like. This can be done throughout the day at breakfast, lunch, and dinner or when you experience a craving.

Theme Mindful eating reminder: Try and take a mindful bite or spoonful for lunch/dinner today. What does it taste like, smell like, feel like, and sound like? Chew the food slowly. Mindfully stop when your body indicates you are full.

Theme: Mindful Exercise: Mindfulness of exercise involves being aware of your body's needs and focusing with mindful attention to your movement. What types of movement did you do and how did it feel? Journal your entry.

Theme : Gratitude : Reflect on what you are grateful for right now in your student life and write everything down.

Theme : Loving Kindness: Do something nice for a student on campus or in class you don't know.

Theme: Mindfulness Motivation: Students find that having a friend check on in you or sending a text to a friend to be mindful is a good way to promote mindfulness and motivate you to truly be present.

Theme: Informal Mindfulness: Subtheme Student Tip: Students recommend having a scavenger hunt with your mates by finding the most beautiful place around you. This can be a good mindfulness exercise by tuning in with all of your senses.

MMS: Inspirational Images:
Mountain Concentration Meditation-Send Himalayas Mountain image: You are the mountain. Breath in strong, breathe out calm. Different seasons, natural, and manmade disasters may affect your surface, but you are whole and strong.

Lake Concentration- send lake image at UQ: You are still like the lake. No matter what is going on within your outer layers, your foundation is still and calm. Breathe these qualities in and out.
Or Forest Fire: You are a forest that grows not matter what the condition. The fire will affect you just as any problem you experience as a student will be felt. But your new seeds plant and you grow again, stronger and more resistant than before. You learn lessons of wisdom as you grow and are more connected with life.
Image of a forest
You are a waterfall. With each flow of turbulent water you remain. Some days the flow is more turbulent than on other days. Regardless, you thrive and continue to remain amidst the turbulent waters and each challenge they bring.

Reference

1. Labee E. Psychology Moment by Moment: A guide to Enhancing Your Practice with Mindfulness Meditation. *New Harbinger Publications.* 2011.
2. Lyzwinski LN, Caffery, L.C., Bambling, M., Edirippulige, S. University Students' Perspectives on a Mindfulness App for Weight Management, Weight related Behaviours, and Stress: A Qualitative Focus Group Study. *American Journal of Health Education.* 2018;9(6).
3. Somov P. Mindful Practices to Overcome Overeating One Meal at a Time. *New Harbinger Publications.* 2008.
4. Stahl BG, Elisha. MBSR Everyday: Daily Practices from the Heart of MBSR. *New Harbinger Publications.* 2015.
