# Supplementary material for: The Mindfulness App Trial for Weight, Weight-Related Behaviors, and Stress in University Students: Randomized Controlled Trial
Source: JMIR Mhealth Uhealth. 2019 Apr 10;7(4):e12210. doi: 10.2196/12210 (PMC6479283; doi:10.2196/12210)
Supplement: Multimedia Appendix 6 [file mhealth_v7i4e12210_app6.pdf]

Consort E-Health Checklist Essentials

Section Topic	Consort Checklist item	EHealth Extensions	Response	Page Number
Title and Abstract	Identification as a RCT	Mentions Primary Condition or Target Group	Yes<br>Yes	RCT on title page And abstract p. 1-2)
Title and Abstract	Structured Summary in Abstract of methods, results, conclusion, trial design	Structured Summary in Abstract of methods, results, conclusion, trial design	Yes	Abstract (p. 2)
Introduction Background and objectives	Scientific Background and Rationale	Describe the problem and solution with rationale	Yes	Introduction (p.3)
Trial Design	Eligibility Criteria	Settings and Location	Yes<br>Yes	Methods section (p. 4)
Interventions	Access	Mode of Delivery<br>Report prompts reminders<br>Describe co-interventions	Yes<br>Yes<br>Yes<br>None	Methods Section (p.4)

Section Topic	Consort Checklist item	EHealth Extensions	Response	Page Number
Outcomes	Clearly defined<br>Pre-specified		Yes clear outcomes	Methods (p 5)
Sample size	Description of how it was calculated		Yes	Methods (p 5)
Randomization	Description		Yes	Methods (p. 4)
Sequence generation	Allocation concealment		Yes	Methods (p.4)
Blinding	Description of who was blinded		Yes	Methods (p.4)
Statistical Methods	Clear Stat methods	Description of analysis methods for attrition	Yes<br>Yes	Methods (p.5)
Ethics	Ethics	Ethics Approval<br>Informed Consent	Yes<br>Yes	Methods (p.5)

Section Topic	Consort Checklist item	EHealth Extensions	Response	Page Number
Numbers analysed	For each group	Report N<br>Effect sizes	Yes	Results (p.6)
Results	Baseline Data	Demographics	Yes<br>Yes	Results (p.7)
Interpretation Principal Findings	Interpretation consistent with results	Restate study questions and summarize answers	Yes	Discussion (p.10-11)
Discussion Limitations	Trial limitations	Typical limitations in e-health trials	Yes	Discussion (limitations) (p.11)

Section Topic	Consort Checklist item	EHealth Extensions	Response	Page Number

Section Topic	Consort Checklist item	EHealth Extensions	Response	Page Number

Section Topic	Consort Checklist item	EHealth Extensions	Response	Page Number

Section Topic	Consort Checklist item	EHealth Extensions	Response	Page Number

Section Topic	Consort Checklist item	EHealth Extensions	Response	Page Number

Section Topic	Consort Checklist item	EHealth Extensions	Response	Page Number

Section Topic	Consort Checklist item	EHealth Extensions	Response	Page Number
